# Safety and Efficacy of Intrapleural Tissue Plasminogen Activator and DNase during Extended Use in Complicated Pleural Space Infections

**DOI:** 10.1155/2016/9796768

**Published:** 2016-06-30

**Authors:** Jason R. McClune, Candice L. Wilshire, Jed A. Gorden, Brian E. Louie, Alexander S. Farviar, Michael J. Stefanski, Eric Vallieres, Ralph W. Aye, Christopher R. Gilbert

**Affiliations:** ^1^Division of Pulmonary, Allergy, and Critical Care Medicine, Penn State Milton S. Hershey Medical Center, Hershey, PA 17033, USA; ^2^Division of Thoracic Surgery and Interventional Pulmonology, Swedish Cancer Institute and Medical Center, Seattle, WA 98104, USA

## Abstract

The use of intrapleural therapy with tissue plasminogen activator and DNase improves outcomes in patients with complicated pleural space infections. However, little data exists for the use of combination intrapleural therapy after the initial dosing period of six doses. We sought to describe the safety profile and outcomes of intrapleural therapy beyond this standard dosing. A retrospective review of patients receiving intrapleural therapy with tissue plasminogen activator and DNase was performed at two institutions. We identified 101 patients from January 2013 to August 2015 receiving intrapleural therapy for complicated pleural space infection. The extended use of intrapleural tissue plasminogen activator and DNase therapy beyond six doses was utilized in 20% (20/101) of patients. The mean number of doses in those undergoing extended dosing was 9.8 (range of 7–16). Within the population studied there appears to be no statistically significant increased risk of complications, need for surgical referral, or outcome differences when comparing those receiving standard or extended dosing intrapleural therapy. Future prospective study of intrapleural therapy as an alternative option for patients who fail initial pleural drainage and are unable to tolerate/accept a surgical intervention appears a potential area of study.

## 1. Introduction

Pleural space infections have been described since the time of Hippocrates [1–4] and unfortunately remain an ongoing problem in modern day healthcare [5–7]. While morbidity and mortality have improved [2], recent data suggests growing concerns related to pleural infection incidence and management [8–11].

Complicated pleural space infections (empyema and complicated parapneumonic effusions) develop in up to two-thirds of those hospitalized with pneumonia, subsequently associated with a 10–20% mortality [6]. While debate regarding surgical versus nonsurgical management exists [5, 12–17], the use of antimicrobials and pleural drainage appears to remain a basic tenement of treatment [6]. While empyema has historically been a surgical disease, more recent randomized clinical trial has suggested the use of intrapleural therapy with a combination of a direct-acting fibrinolytic agent (recombinant tissue plasminogen activator) and a hydrolytic enzyme of DNA (deoxyribonuclease) improves radiographic and clinical outcomes (including the need for surgical referral) in patients with complicated pleural space infections [18]. This protocol utilizes six doses of tissue plasminogen activator (t-PA) and DNase over 72 hours; however, to our knowledge the efficacy and safety profile of extended use of this therapy remains unknown. We sought to describe the safety profile and outcomes of extended (>6 consecutive doses) use in adult patients with complicated pleural space infections.

## 2. Methods

A retrospective review of all patients receiving t-PA and DNase at two institutions (Swedish Medical Center and Penn State Milton S. Hershey Medical Center) was performed, from January 2013 to August 2015. The institutional review board at both institutions approved this study (Penn State Milton S. Hershey Medical Center, STUDY00002820, and Swedish Medical Center, 5845S-15) and the requirement for informed consent for data collection and analysis was waived.

Pharmacy databases were queried for intrapleural use of recombinant human DNase (Pulmozyme, Roche) and recombinant tissue plasminogen activator (Actilyse, Boehringer Ingelheim). Inclusion criteria for analysis included (1) confirmed/suspected empyema or complicated parapneumonic effusion undergoing tube thoracostomy drainage, (2) inpatient use of IPFT in consecutive fashion (totaling greater than 3 doses), and (3) age > 18 years. Exclusion criteria included previous pleural interventions (other than antibiotics and tube thoracostomy placement) for infection or an incomplete medical record.

As all patients undergoing t-PA and DNase therapy were queried, we collected data on patients who underwent standard (6 doses or less) as well as those who underwent extended (>6 doses) intrapleural therapy. These groups represented unmatched populations from the same time frame, all treated for complicated pleural space problems with t-PA and DNase intrapleural therapy. Medical records were queried for demographics, comorbidities, radiographic findings, pleural fluid characteristics, intrapleural therapy, and clinical outcomes. Complicated pleural space infection was defined as (1) empyema, in which macroscopic pus or the presence of bacteria was identified on gram stain and/or culture, or (2) suspected pleural infection, patient with clinical evidence of infection and pleural fluid biochemical profile consistent with complicated parapneumonic infection [6, 19]. Medical records were further queried for evidence of intrapleural therapy complications defined as (1) a bleeding event requiring administration of packed red blood cells, during or within 72 hours of t-PA and DNase administration, (2) inadvertent chest tube dislodgement, (3) hospital readmission within 30 days of initial event, (4) placement of additional chest tube(s), (5) discharge from the hospital with ongoing pleural drainage, and (6) new or escalating narcotic requirements.

Both centers participated in data collection and offer multidisciplinary care in thoracic and respiratory disease. No specific protocol was utilized, with the suitability and timing of t-PA and DNase intrapleural therapy determined by the attending physician. However, general practice at both institutions has been reflective of the MIST II protocol [18], intrapleural delivery of tissue plasminogen activator (10 mg) and DNase (5 mg) twice daily, totaling six doses. The indication and utilization of extended intrapleural dosing were at the discretion of the attending physician.

Study data were collected and managed using REDCap electronic data capture tools hosted at the Penn State Milton S. Hershey Medical Center. Statistical analysis was conducted using Microsoft Excel, version 14.4.2 (Microsoft Corporation, Redmond, WA, USA) and SPSS 19.0 (SPSS Inc., Chicago, IL, USA). Continuous variables were summarized using mean and median values with range and interquartile ranges. Comparisons were made using the Mann-Whitney test. Categorical variables were summarized using counts and percentages. Groups were compared using the Yates chi-square test or Fisher's exact test when any of the subgroups had five or less components. A *p* < 0.05 was considered significant.

## 3. Results

**Figure 1:**
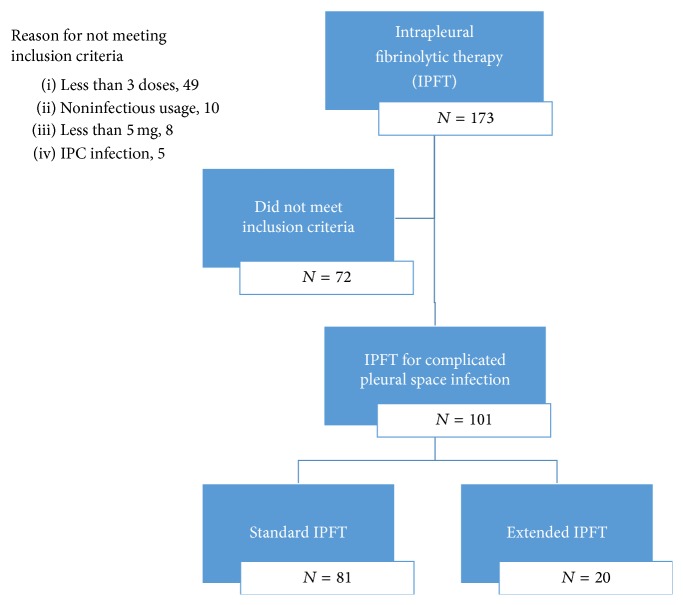
Patient exclusion/inclusion criteria.

The use of t-PA and DNase from January 2013 to August 2015 was identified in 173 patients. A total of 72 patients received t-PA and DNase but did not meet the inclusion criteria (Figure 1), leaving a total of 101 eligible patients. No patients were excluded due to incomplete medical records or previous pleural interventions. Baseline characteristics and demographics for the 101 patients included in the final analysis are displayed in Table 1. Many baseline characteristics of the groups were similar; however the presence of hospital acquired infection and fluid culture positivity was more prevalent within the group undergoing extended intrapleural therapy.

Extended dosing of intrapleural therapy was identified in 20% (20/101) of patients. The mean number of t-PA and DNase doses in those undergoing extended dosing was 9.8 (range of 7–16). The median age was 57 (IQR: 49–64) years with a slight male predominance (12/20). Outcome data for both standard and extended groups are available in Table 2. Although there appeared to be an overall trend to longer lengths of pleural drainage, hospital stay, and complications when comparing the standard to extended groups, none reached statistical significance. The overall referral for surgical intervention appeared similar (16% versus 15%). Trends towards more bleeding events (2.5% versus 10%), additional chest tube placement (14.8% versus 35%), and requirements for new or escalating doses of narcotics (56.8% versus 80%) all appeared within the group undergoing extended intrapleural therapy; however none reached statistical significance (*p* = 0.365, 0.080, and 0.056, resp.).

## 4. Discussion

Herein, we present data on the use of extended intrapleural therapy with t-PA and DNase, suggesting that it may possess an acceptable safety and outcome profile for the treatment of complicated pleural space infections. Within our study population, surgical referral and complication rates appear comparable between the use of an extended dosing regimen versus “standard” therapy and other historical control populations [18, 20].

The use of intrapleural therapies for treating complicated pleural space infections has been investigated for over 25 years with mixed results [20–24]. The MIST II trial [18] has demonstrated an improvement in radiographic evidence of pleural disease but also a 77% reduction in initial surgical referral within the combination therapy group [18]. This finding may represent a potential paradigm shift of empyema to a nonsurgical disease, being further supported by an additional observational trial reporting the avoidance of surgical intervention in complicated pleural space infections in over 92% of their selected population when managed with t-PA and DNase [25].

Many questions regarding intrapleural therapy and its utilization still exist, including timing of dosing and strength of dosing. Studies currently exist supporting use of extended dosing for fibrinolytic monotherapy such as streptokinase and urokinase [23, 26, 27], oftentimes on a daily basis. At the current time, we are unable to find literature supporting t-PA and DNase use after the initial dosing period, an “extended” dosing period.

We propose that the use of an extended dosing regimen of t-PA and DNase may offer an alternative therapeutic option in patients that are unfit or refuse surgical intervention when demonstrating evidence of persistent pleural space infection. While many textbooks and surgeons often consider empyema a “surgical disease,” the role of surgical management in empyema continues to be debated [14, 15, 17, 28–30]. While neither Rahman et al. [18] nor Piccolo et al. [25] included/excluded patients within their studies based on surgical candidacy, it remains unclear who would have actually been surgical candidates and data suggests that patients undergoing surgical intervention are often “selected” on the basis of younger age and lower comorbidity indexes [8]. This bias may exclude a large population unable to receive surgical intervention, for which prolonged intrapleural therapy with t-PA and DNase may offer a potential alternative.

While our subset of patients appear similar in many demographic measurements, we must acknowledge that patients presenting with complicated pleural space infections are likely a fairly heterogenous population and when studied in a nonrandomized fashion such as this concern for selection bias must exist, a fact likely present within the Piccolo et al. [25] population as well. Hospital acquired infection and pleural culture positivity appear more prevalent within those undergoing extended intrapleural therapy; however no other significant differences, including the RAPID pleural infection score [31], were identified (Table 1). Due to the retrospective nature of our study it was not possible to control which patients received extended dosing versus standard dosing, introducing a selection bias within our population. While this represents a significant selection bias, it also allows us to represent current clinical care of complicated pleural space infections. Selection bias for those receiving tube thoracostomy drainage, intrapleural therapy, and surgical treatment for empyema was unable to be accounted for as we did not track all patients with suspected or confirmed complicated pleural infection. Utilizing our search criteria we were unable to identify patients not undergoing treatment, those undergoing successful tube thoracostomy drainage, or those undergoing surgery. Anecdotally, these numbers would be small, as both institutions employ multidisciplinary teams of interventional pulmonology and thoracic surgery that essentially follow all complicated pleural space infections admitted to the hospital.

In general practice, extended intrapleural dosing is utilized for patients displaying persistent evidence of pleural space infection despite tube thoracostomy drainage and six doses of t-PA and DNase. However, due to the retrospective data collection process we cannot confirm that ongoing pleural infection was indeed the main indication of extended intrapleural dosing use and/or surgical referral, a limitation of our study. Medical records were reviewed to confirm the indications for proceeding; however the potential for inaccuracy must be acknowledged. Incomplete medical records were minimized through use of the electronic medical record at both institutions. Appropriate patient follow-up was maximized as it was limited to care at hospital discharge or short-term ambulatory follow-up (if ambulatory drainage was required), allowing for our observed follow-up rate of 100%. However, if patients were admitted at another facility we may not have captured that data, potentially altering our ultimate outcome and complication rates.

Within the extended use group we identified some trends towards longer hospital stays and longer chest tube days; however none were statistically significant. This phenomenon was also observed within the complications observed, such as bleeding, chest tube dislodgement, and increased narcotic use. However, due to the small sample size, particularly within the extended dosing group, our ability to draw conclusions must remain tempered. This limitation must also be recognized when comparing adverse event profiles due to the concern for potential type II error. However, as previously described, adverse events were observed within both groups with no statistical difference between groups (Table 2). While bleeding complications may be the most feared when utilizing an antithrombotic medication like t-PA, within our population bleeding events occurred with fairly similar frequency in both the standard and extended dosing regimens and with similar frequency to previously described reports [18, 20, 25, 26].

In conclusion, we present data on the use of extended dosing with t-PA and DNase in patients with complicated pleural space infections. Within the population studied there appears to be no statistically significant increased risk of complications, surgical referral, or outcome differences when compared to those receiving “standard” intrapleural therapy or to historical controls. The use of extended therapy as an alternative option for patients who fail initial standard therapy and are unable to tolerate/accept a surgical intervention for their complicated pleural space infection appears to be a potential area of study.

## Figures and Tables

**Table 1:** Demographics of patients receiving intrapleural therapy.

	Standard IPFT	Extended IPFT	*p* value
Patients (*n*)	81	20	
Age (years)			
Median [interquartile range (25–75)]	62 [44–74]	57 [49–64]	0.394
Sex (*n*)			0.648
Male	44	12	
Female	37	8	
Body mass index (kg/m^2^)			
Median [interquartile range (25–75)]	25 [22–31]	22 [21–25]	0.097
RAPID pleural infection score			
Mean [standard deviation]	3.0 [1.5]	2.7 [1.4]	0.564
Etiology of infection (*n*, (%))			0.616
Primary	64	16	
Bacteremia	8	3	
Postthoracic surgery	9	0	
Other	0	1	
Hospital acquired infection (*n*, (%))	18 (22)	10 (50)	0.013
Median pleural fluid characteristics [interquartile range (25–75)]			
Lactate dehydrogenase (units/L)	1118 [541–4117]	827 [484–6261]	0.980
Protein (g/dL)	4 [3-4]	3 [3-4]	0.561
Glucose (mg/dL)	69 [25–92]	57 [32–81]	0.652
Pleural fluid microbiology analysis			
Gram stain, positive (*n*, (%))	27 (33)	8 (40)	0.575
Fluid culture, positive (*n* (%))	29 (36)	15 (75)	0.002

**Table 2:** Outcomes of patients receiving intrapleural therapy.

	Standard IPFT	Extended IPFT	*p* value
Patients (*n*)	81	20	
Total number of chest tubes [median [interquartile range (25–75)]]	1 [1-1]	2 [1-2]	0.015
Placement of additional chest tube after IPFT initiation (*n* (%))	12 (15)	7 (35)	0.080
Median outcomes in hospital days [interquartile range (25–75)]			
Chest tube duration	6 [4–11]	8 [6–11]	0.200
Hospital length of stay	13 [9–19]	17 [9–25]	0.355
Admission to lytic cessation	7 [5–11]	9 [5–12]	0.281
Admission to surgery day	6 [0–14]	7 [7–14]	0.975
Day of surgery to discharge	11 [6–21]	16 [11–22]	0.534
Lytic cessation to discharge	4 [3–9]	3 [1–19]	0.404
Referral for surgery (*n* (%))	13 (16)	3 (15)	0.821
Complications (*n*, (%))			
Readmission	13 (16)	2 (10)	0.741
Outpatient pleural drainage	10 (12)	2 (10)	0.924
Bleeding	2 (3)	2 (10)	0.365
Tube dislodgement	3 (4)	3 (15)	0.166
New narcotic use	46 (57)	16 (80)	0.056
